# *O*^6^-alkylguanine-DNA Alkyltransferases in Microbes Living on the Edge: From Stability to Applicability

**DOI:** 10.3390/ijms21082878

**Published:** 2020-04-20

**Authors:** Rosanna Mattossovich, Rosa Merlo, Riccardo Miggiano, Anna Valenti, Giuseppe Perugino

**Affiliations:** 1 Institute of Bioscience and BioResources, National Research Council of Italy, Via Pietro Castellino 111, 80131 Naples, Italy; rosanna.mattossovich@ibbr.cnr.it (R.M.); rosa.merlo@ibbr.cnr.it (R.M.); 2 Department of Pharmaceutical Sciences, University of Piemonte Orientale, Via Bovio 6, 28100 Novara, Italy; riccardo.miggiano@uniupo.it

**Keywords:** thermophilic sources, DNA repair, biotechnological tools, alkylation damage, AGT

## Abstract

The genome of living cells is continuously exposed to endogenous and exogenous attacks, and this is particularly amplified at high temperatures. Alkylating agents cause DNA damage, leading to mutations and cell death; for this reason, they also play a central role in chemotherapy treatments. A class of enzymes known as AGTs (alkylguanine-DNA-alkyltransferases) protects the DNA from mutations caused by alkylating agents, in particular in the recognition and repair of alkylated guanines in *O*^6^-position. The peculiar irreversible self-alkylation reaction of these enzymes triggered numerous studies, especially on the human homologue, in order to identify effective inhibitors in the fight against cancer. In modern biotechnology, engineered variants of AGTs are developed to be used as *protein tags* for the attachment of chemical ligands. In the last decade, research on AGTs from (hyper)thermophilic sources proved useful as a model system to clarify numerous phenomena, also common for mesophilic enzymes. This review traces recent progress in this class of *thermozymes*, emphasizing their usefulness in basic research and their consequent advantages for in vivo and in vitro biotechnological applications.

## 1. Introduction

**Figure 1:**
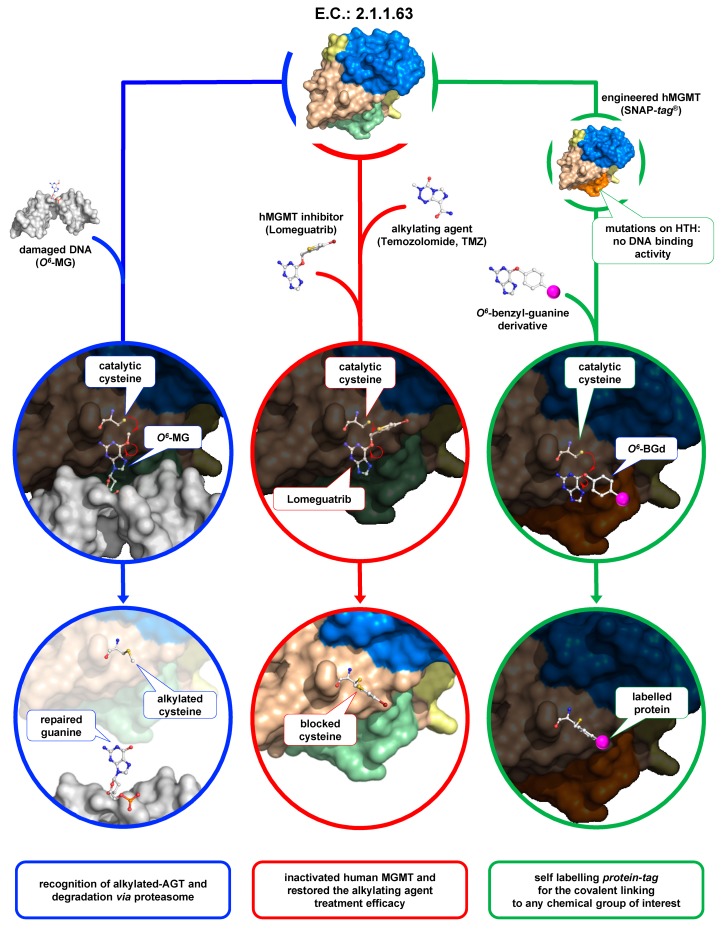
The *O*^6^-alkyl-guanine-DNA-alkyl-transferase (AGT)s’ world. AGTs are small enzymes composed by a N-terminal domain (in sky blue), a C-terminal domain (in light brown) connected by a loop (in yellow). In the C-terminal domain, a helix-turn-helix motif (in light green, or in orange in the SNAP-*tag*) is responsible of the DNA-binding activity. The peculiar irreversible reaction mechanism of these enzymes plays a pivotal role in the physiological DNA repair (blue path), and it has important repercussions in cancer cell treatment (red path) and biotechnological applications (green path). Atoms are coloured by the CPK colour convention.

Monofunctional alkylating agents, a class of mutagenic and carcinogenic agents present in the environment, induce DNA alkylation in several positions including guanine at *O*^6^ (*O*^6^-MG; 6% of adducts formed), the *N*^7^ of guanine (*N*^7^-MG; 70%), and the *N*^3^ of adenine (*N*^3^-MA; 9%) [1]. Alkylation of guanine (*O*^6^-AG) is a cytotoxic lesion, although the specific mechanism of this cytotoxicity is not yet fully understood. It was proposed that the toxic effect occurs after DNA replication, because the *O*^6^-AG incorrectly base-pairs with thymine generating a transition from G:C to A:T [2]. The mutations caused by *O*^6^-MG that occur at the time of replication are recognized by the post-replication mismatch repair system with potential harmful implications for cell viability. Apart from conventional DNA repair pathways as Mismatch Excision Repair (MMR), Nucleotide Excision Repair (NER), Base Excision Repair (BER), alkylated-DNA protein alkyl-transferases (called *O*^6^-alkyl-guanine-DNA-alkyl-transferase (AGT or OGT) or *O*^6^-methyl-guanine- DNA-alkyl-transferase (MGMT); EC: 2.1.1.63) perform the direct repair of alkylation damage in DNA [3,4]. They represent the major factor in counteracting the effects of alkylating agents that form such adducts [4]. These are small enzymes (17–22 kDa) that are widely present in organisms of the three kingdoms (bacteria, archaea, eukaryotes) but apparently absent from plants, *Schizosaccharomyces pombe*, *Thermus thermophilus*, and *Deinococcus radiodurans*. The reaction mechanism of AGTs is based on the recognition of the damaged nucleobase on DNA [5], followed by a one-step SN_2_-like mechanism, in which the alkyl group of the damaged guanine is irreversibly transferred to a cysteine residue in its active site [5,6,7,8] (Figure 1, blue path).

For these reasons, they are also called *suicide* or *kamikaze* proteins, showing a 1:1 stoichiometry of their reaction with the natural substrate. The disadvantage of this elegant catalysis is that, upon alkylation, the protein is self-inactivated and destabilized, triggering its recognition by cellular systems to be degraded by the proteasome [8,9].

### 1.1. AGTs as Targets in Cancer Therapy 

Alkylation damage to DNA occurs in various living conditions, and for this reason the widespread presence of AGT protects cells from killing by alkylating agents. However, human AGT (hMGMT) is a *double-edged* sword—on the one hand, it protects healthy cells from these genotoxic and carcinogenic effects, but also counteracts alkylating agents-based chemotherapy by protecting cancer cells from the killing effect of these drugs [10,11]. Consequently, hMGMT has emerged as a crucial factor in anticancer therapies [12]—an inverse relationship has been discovered between the presence of hMGMT and the sensitivity of cells to the cytotoxic effects of alkylating agents, such as temozolomide (TMZ), in different types of cancer cells, including prostate, breast, colon, and lung cancer cells [13].

The resistance to chemotherapy may be reduced by inhibition of these enzymes; as described before, after removing the lesion, the alkylated form of the protein is inactivated and enters the intracellular degradation pathways. Hence, in order to counteract the action of hMGMT in chemotherapy regimens, a large number of studies aimed to the develop hMGMT inhibitors to be used in combination with alkylating agents. In view of this therapeutic relevance, much success has been obtained through the design of hMGMT pseudo-substrates, namely, the *O*^6^-benzylguanine (*O*^6^-BG) and the strong inactivator *O*^6^-[4-bromothenyl]-guanine (*O*^6^-BTG, Lomeguatrib) [13,14]. These compounds mimic damaged guanine on DNA and react with the protein by the covalent transfer of the alkyl adduct to the active site cysteine residue, thus irreversibly inactivating the enzyme (Figure 1, red path). Therapeutically, *O*^6^-BG is not toxic on its own, but renders cancer cells 2 to 14 times more sensitive to alkylating agents’ effects. The oligonucleotides containing several *O*^6^-BG are potent inhibitors and represent a valid alternative to the use of free modified guanines, thereby improving the activity of the alkylating chemotherapy drug in the treatment of some tumours [15,16,17].

### 1.2. AGTs and Biotechnology

The specific labelling of proteins with synthetic probes is an important advance for the study of protein function. To achieve this, the protein of interest is expressed in a fusion with additional genetically encoded polypeptides, called *tags*, which mediate the labelling. The first example of an *autofluorescent tag* was the *Aequorea victoria* green fluorescent protein (GFP) allowing the in vivo localization of fusion proteins in cellular and molecular biology [18,19]. Among *affinity tags*, of particular importance are the poly(His)-*tag*, the chitin-binding protein, the maltose-binding protein [20], the Strep-*tag* [21], and the glutathione-S-transferase (GST-*tag*) [22], which allow fast and specific purification of proteins of interest from their crude biological source using affinity techniques. *Solubilization tags* are especially useful to assist the proper folding of recombinant proteins expressed in chaperone-deficient species such as *Escherichia coli*, avoiding protein precipitation and the use of alternative expression protocols [23,24]—these include thioredoxin [25] and poly(NANP). 

However, all the *tags* listed above are limited by the fact that each of them can be used for one or only a few applications. The need therefore emerged to develop a *universal tag* that could widely cover several applications.

In 2003, the group headed by Kai Johnsson pioneered the use of an engineered hMGMT variant as a fusion protein for in vitro and in vivo biotechnology applications, which led then to its commercialization, namely, the SNAP-*tag* (New England Biolabs) [26,27,28,29]. They started from the knowledge that hMGMT tolerates the presence of groups conjugated to the pseudo-substrate *O*^6^-BG (*O*^6^-BG derivatives)—the unusual covalent bond with the benzyl moiety can therefore be exploited for “biotech” purposes (Figure 1, green path). Thanks to its small size, the engineered hMGMT (SNAP-*tag*) can be fused with other proteins of interest. The expression of the fusion protein inside the cells followed by incubation with opportune fluorescent derivatives leads to in vivo labelling of fusion proteins with the probe, which can be used for localization studies [26]. The same principle has also been used for the immobilization of tagged fusion proteins in vitro [30]. This offers a delicate condition for fixing and disposing in a better orientation of a wide range of proteins/enzymes on a surface. The SNAP-*tag* technology was successfully applied to surface plasmon resonance (SPR) for the covalent immobilization of proteins of interest [31]. Another interesting application of this protein-tag is the possibility to produce new antibody fragments (scFv-SNAP) to be employed in the SPR analysis [32].

Despite the need to use a specific substrate, SNAP-*tag* offers endless applications—the possibility to covalently link a desired chemical group (conjugated to the *O*^6^-BG) to a protein of interest (genetically fused to it) makes it decidedly advantageous, if compared to traditional *protein tags* currently in use. Table 1 shows a brief comparison between some examples of *protein tags* and the SNAP-*tag* in several application fields.

## 2. Thermophilic and Thermostable AGTs

As for organisms living under mesophilic conditions, environmental and endogenous alkylating agents also attack the genome of thermophilic and hyper-thermophilic organisms. Additionally, high temperatures accelerate the process of alkylation, leading to DNA breaks [33], because alkylating agents are chemically unstable at the physiological conditions of these organisms, and their collateral decomposition may worsen the formation of DNA alkylation products [34]. Thus, the presence of AGTs and methylpurine glycosylases in hyperthermophilic organisms implies that they are naturally exposed to endogenous methylating agents [34], thus supporting the crucial role of AGTs [35,36]. 

**Figure 2:**
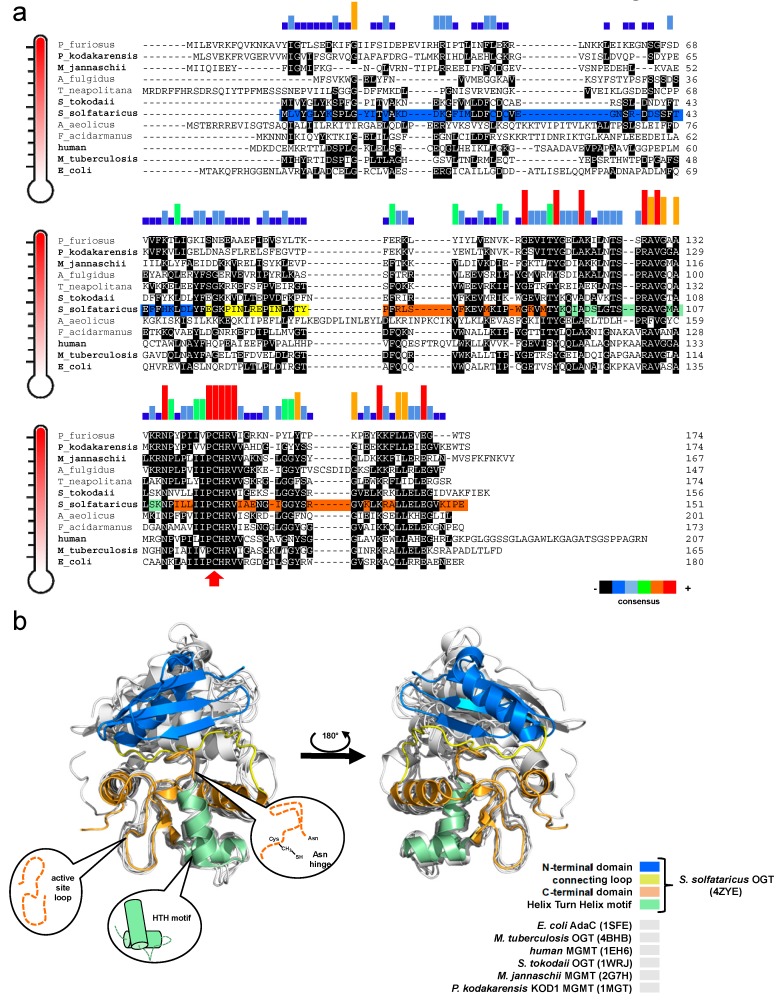
(**a**) Alignment of biochemically and structurally (in bold) characterized AGTs. DNA sequences are listed in decreasing order of temperature. The histograms in different colours show the sequence consensus, and the red arrow indicates the highly conserved catalytic cysteine. (**b**) Superimposition of all known AGT structures in their free form (in grey). All common domains and elements are coloured only for the *O*^6^-alkyl-guanine-DNA-alkyl-transferase protein from the archaeon *Saccharolobus solfataricus* (hereinafter *Ss*OGT) enzyme. Coloured bars behind the *Ss*OGT sequence in (**a**) recall the enzyme domains highlighted in the structure and in the legend in (**b**).

Apart from some studies on Archaea using cell-free extracts, a few examples of biochemical studies of AGTs from thermophilic sources include the enzymes from *Pyrococcus* sp. KOD1 [35] conducted by Imanaka and co-workers from *Aquifex aeolicus* and *Archaeoglobus fulgidus* performed by the group of Prof. Pegg in 2003 (Figure 2) [34]. Intriguingly, *A. aeolicus* AGT, whose organism was identified as the most primitive bacterium, is closer to the mammalian AGTs than other bacterial homologues in terms of *O*^6^-BG sensitivity [34].

### The Common Themes in AGTs’ Tertiary Structure and the Intrisic Factors of Stability

Despite the different primary structures (Figure 2a), thermophilic enzymes show a typical AGT protein architecture, consisting of two domains [37]: a highly conserved C-terminal domain (CTD), surprisingly superimposable for all available AGT structures (Figure 2b), and a N-terminal domain (NTD), which is very different among AGTs and whose function is not well understood (likely involved in regulation, cooperative binding, and stability [6,38,39]). The CTD contains the DNA binding *helix-turn-helix* motif (HTH); the *Asn hinge*, which precedes the -V/IPCHRVV/I- amino acid sequence containing the conserved catalytic cysteine (except the *Caenorhabditis elegans* AGT-2 that has the -PCHP- sequence [40,41]); and the *active site loop*, responsible for the substrate specificity. 

A comparative structural analysis performed on AGT proteins whose structures are in the Protein Data Bank revealed significant differences of the intrinsic structural features that have been considered to be relevant for thermostability, such as helix capping, intramolecular contacts (hydrogen bonds, ion-pairs), and solvent-accessible surface areas. Helix capping plays a central role in the stability of α-helices, due to lack of intra-helical hydrogen bonds in the first and last turn [42,43], and its effect results in an overall structural stabilization of protein folding [44]. By inspecting the crystal structure of *Ss*OGT (PDB ID: 4ZYE), considered here as the thermophilic reference AGT protein, we verified that the five α-helices of the composing the protein tertiary structure are characterized by the presence of helix capping, this possibly increasing the thermal stability. In particular, the helix *H1* at the NTD is stabilised by a peculiar double serine sequence (S40–S41) and a glutamic acid (E54) at its CTD, the latter is strictly conserved in all AGTs from thermophilic organisms (see Figure 2a). The HTH motif, built on helices *H3* and *H4*, is stabilized at the level of *H3* by a highly conserved threonine residue (T89) as N-cap and a serine (S96), distinctive of *Ss*OGT, as C-cap. Furthermore, helix *H4* contains two serine-based capping among which the one placed at NTD (S100) is strictly conserved in all thermophilic AGTs and is followed by a proline (P101) that fits well in the first turn of the helix thanks to its own backbone conformation. Finally, the helix *H5* is protected by glutamic acid capping that is present in all the AGTs from different species. Another feature contributing to thermal stability is the solvent-accessible surface area (SASA). Indeed, the decrease of SASA and the increase of hydrophobic residues that are buried from the solvent have stabilizing principles for thermostable protein [45]. As described in Table 2, *Ss*OGT shows the smaller total SASA value, in line with its exceptional stability. On the contrary, OGT from *Mycobacterium tuberculosis* [38,46] has a higher value due to the peculiar conformation of both the active site loop and the C-terminal tail that are exposed to the bulk solvent and are less heat stable (Table 2).

Finally, by comparing hyperthermophilic AGTs with the orthologs from mesophilic organisms, in terms of atomic contacts between charged residues as well as intramolecular hydrogen bonds (Table 2), significant differences emerged in the number of charged residues contacts. As expected for thermostable proteins [47], *Ss*OGT, as well as the proteins from *Sulfurisphaera tokodaii* and *Pyrococcus kodakaraensis*, shows a larger number of electrostatic contacts, characterized by higher bond-dissociation energy, with respect to hydrogen bonds for which we did not detect significant differences among the analysed structures, apart from MGMT of *P. kodakaraensis* (*Pk*-MGMT) [48].

Although the number of H-bonds is approximately similar across the AGTs from different organisms, there should be differences in the position-related role of such bonds, supporting overall stability of thermophilic variants. With reference to *Pk*-MGMT, Hashimoto and co-workers detected the same number of ion-pairs between the extremophilic protein and *E. coli* Ada-C [49]; however, more intra- and inter-helix ion pairs were found in *Pk*-MGMT. Although the absence of a correlation between ion pairs’ position and stabilization in Ada-C exists, the intra-helix ion pairs act in the secondary structure of *Pk-MGMT*, stabilizing helices, and the inter-helix ion pairs consolidate the inter-domain interactions, enhancing the stability of the tertiary structure packing. 

## 3. The *O*^6^-Alkylguanine-DNA-Alkyltransferase from *Saccharolobus solfataricus*

In the last decade, *Ss*OGT has been characterized through detailed physiological, biochemical, and structural analysis. Due to its intrinsic stability, the *Ss*OGT protein has proven to be an outstanding model for clarifying the relationships between function and structural characteristics. 

*Saccharolobus solfataricus* (previously known as *Sulfolobus solfataricus*) is a microorganism first isolated and discovered in 1980 in the Solfatara volcano (Pisciarelli-Naples, Italy) [51], which thrives in volcanic hot springs at 80 °C and a pH 2.0–4.0 range. In order to protect its genome in these harsh conditions, *S. solfataricus* evolved several efficient protection and repair systems [33,52]. *S. solfataricus* is highly sensitive to the alkylating agent methyl methane sulfonate (MMS), showing a transient growth arrest when treated with MMS concentrations in the range of > 0.25 mM to 0.7 mM [33,52]. Interestingly, although the *ogt* RNA level increases after MMS treatment, the relative enzyme concentration decreases, suggesting its degradation in cells in response to the alkylating agent and, in general, to a cellular stress [52]. Under these treatment conditions, however, the protein level rises after few hours, and, in parallel, the growth of *Saccharolobus* starts again [52], indicating a role of *Ss*OGT in efficient DNA repair by alkylation damage.

### 3.1. Innovative OGT Assays

Various assays to measure AGT activity are reported in the literature. The first methods were based on the use of oligonucleotides carrying radioactive (^3^H or ^14^C) *O*^6^-alkylguanine groups. Proteinase K digestion was then carried out to measure the levels of marked S-methyl-cysteine in the lysate in an automatic amino acid analyser [53]. A very similar, but simpler and faster radioactive assay was used in another procedure with a ^32^P-terminal labelled oligonucleotide containing a modified guanine in a methylation-sensitive restriction enzyme sequence (as *Mbo* I). The AGT DNA repair activity thereby allowed the restriction enzyme to cut [54]. This procedure was also used by Ciaramella’s group to identify for the first time the activity of *Ss*OGT [52]. This test has the advantage of analysing the digested fragment directly by electrophoresis on a polyacrylamide gel [55].

It was therefore improved in terms of precision by the subsequent separation of the digested oligonucleotides by HPLC. The chromatographic separation allowed the calculation of the concentration of active AGT after measuring the radioactivity of the peak corresponding to the digested fragment [55]. Similarly, Moschel’s group developed the analysis of hMGMT reaction products based on HPLC separation in 2002. This test investigated the degree of inhibition of oligonucleotides with *O*^6^-MG or *O*^6^-BG in different positions that varied from the 3’ to the 5’ end and whether they could be used as chemotherapy agents. IC_50_ values were obtained by quantifying the remaining active protein after the radioactive DNA reaction [56].

Although the assay measures the protein activity, the use of radioactive materials and chromatographic separations made these assays long, tedious, and unsafe.

An alternative approach was proposed in 2010 by the group of Carme Fàbrega, who set up an assay based on the thrombin DNA aptamer (TBA), a single-stranded 15 mer DNA oligonucleotide identified via Systematic Evolution of Ligands by EXponential enrichment (SELEX), which in its quadruplex form binds thrombin protease with high specificity and affinity [57]. In this assay, they put a fluorophore and a quencher to the TBA—the quadruplex structure of this oligonucleotide is compromised if a central *O*^6^-MG is present, preventing the two probes to stay closer. An AGT’s repair activity on the oligonucleotide allows the folding of the quadruplex structure and the Förster Resonance Energy Transfer (FRET) energy transfer takes place, resulting in a decrease of the fluorescence intensity [58].

**Figure 3:**
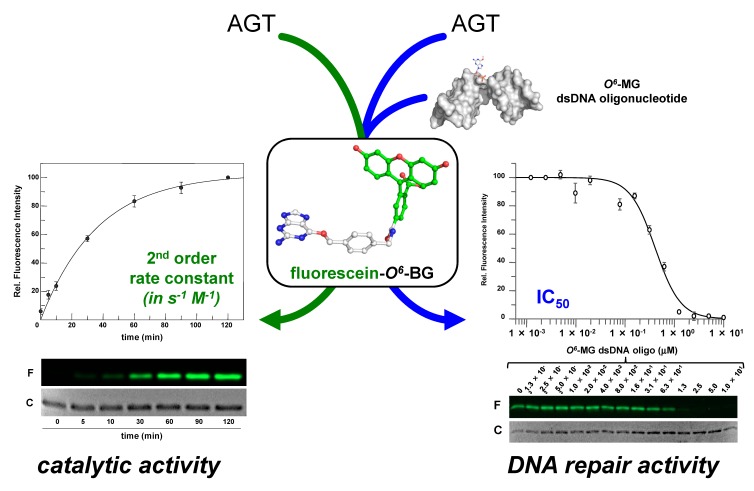
Innovative fluorescent AGT assay. The substrate could be used alone for the determination of the AGT catalytic activity, or in combination with a competitive non-fluorescent substrate (alkylated-DNA). In the latter case, an indirect measure of the DNA repair activity on natural substrates is determined (adapted from [63]).

Recently, the introduction of fluorescent derivatives of the *O*^6^-BG (as SNAP Vista Green, New England Biolabs) made possible the development of a novel DNA alkyl-transferase assay. Because AGT covalently binds a benzyl-fluorescein moiety of its substrate after reaction, it is possible to immediately load the protein product on a SDS-PAGE—the *gel-imaging* analysis of the fluorescence intensity gives a direct measure of the protein activity because of the 1:1 stoichiometry of protein/substrate (Figure 3). Signals of fluorescent protein (corrected by the amount of loaded protein by Coomassie staining analysis) obtained at different times are plotted, and a second order reaction rate is determined [38,39,46,52,59,60]. This method can be applied to all AGTs that bind *O*^6^-BG, with the exception of the *E. coli* Ada-C [61,62]. 

Furthermore, an alkylated double strand DNA (dsDNA) oligonucleotide can be included in a competition assay with the fluorescein substrate. This non-fluorescent substrate lowers the final fluorescent signal on *gel imaging* analysis, depending on its concentration. In this way, it is possible to measure the activity of AGTs for their natural substrate, giving an indirect measure of methylation repair efficiency (Figure 3) [38,39,46,52,59,60]. By using this methodology, it was even possible to discriminate the *Ss*OGT activity regarding the position of the *O*^6^-MG on DNA (see below; [39]), in line with previous data on hMGMT [64].

### 3.2. Biochemical Properties of S. solfataricus OGT

The recombinant *Ss*OGT protein, heterologously expressed in *E. coli*, has been fully characterized using the fluorescent assay described and summarized in Section 3.1, and some results are compiled in Table 3. In agreement with its origin, the protein showed optimal catalytic activity at 80 °C, although retaining a residual activity at lower temperatures (Table 3), and in a pH range between 5.0 and 8.0. As for the most part of many thermophilic enzymes, *Ss*OGT is resistant over a wide range of reaction conditions, such as ionic strength, organic solvents, common denaturing agents, and proteases [52,59]. Interestingly, chelating agents do not affect the activity of this enzyme. Crystallographic data clarified this observation, as the archaeal enzyme lacks a zinc ion in the structure [39], whereas this ion is important for correct folding of hMGMT [6].

### 3.3. Crystal Structure of SsOGT

All catalytic steps of the AGTs’ activity (alkylated DNA recognition, DNA repair, irreversible trans-alkylation of the catalytic cysteine, recognition, and degradation of the alkylated protein) have been structurally characterized. Most information comes from the classic studies on hMGMT, as well as the Ada-C and OGT from *Escherichia coli* [5,6,7,8,49]. Other AGTs’ structures are also available in the Protein Data Bank site (Figure 2a) (http://www.rcsb.org/pdb/results/results.do?tabtoshow=Current&qrid=D3B02F3B).

As shown in Figure 1, all AGTs are inactivated after the reaction and degraded via proteasome, whereas in higher organisms, the degradation is preceded by protein ubiquitination [9]. It is a common view that the recognition of alkylated-AGTs is due by a conformational change; however, data on structure and properties of alkylated AGTs are limited because alkylation greatly destabilizes their folding [39]. The methylated-hMGMT and benzylated-hMGMT 3D structures were only obtained by flash-frozen crystals, showing that alkylation of the catalytic cysteine (C145) induces subtle conformational changes [6,7,65]. Consequently, these structures might not reflect the physiological conformation of the alkylated hMGMT [39].

Concerning the interactions with the DNA, *Ss*OGT binds methylated oligonucleotides. However, the repair activity depends on the position of the alkyl-group [39]. To efficiently repair the alkylated base on DNA double helix, the protein requires at least three bases from either the 5′ or the 3′ end. This is due to the necessary interactions formed with the double helix. Structural analysis confirmed these data [39].

**Figure 4:**
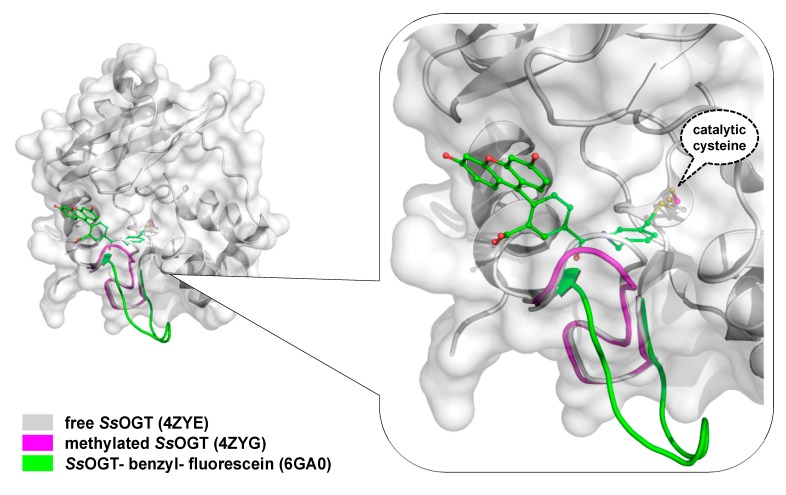
Conformational changes of the *Ss*OGT *active-site loop* after reaction with an *O*^6^-MG dsDNA oligonucleotide (in magenta; [39]), or with SNAP Vista Green substrate (in green; [66]).

To overcome the serious limitation to obtain structural data from mesophilic AGTs after reaction, studies have moved to thermostable homologues, based also on the knowledge that all AGTs share a common CTD domain structure (Figure 2b). In contrast to the human counterpart, alkylated *Ss*OGT was soluble and relatively stable, thus allowing *in-deep* analysis of the protein in its post-reaction form [39]. Structural and biochemical analysis of the archaeal OGT, as well as after the reaction with a bulkier adduct in the active site (benzyl-fluorescein; [66]), suggested a possible mechanism of alkylation-induced *Ss*OGT unfolding and degradation (Figure 4). 

On the basis of their data, Perugino and co-workers suggested a general model for the mechanism of post-reaction AGT destabilization—the so called *active-site loop* moves towards the bulk solvent as a result of the covalent binding of alkyl adduct on the catalytic cysteine and the extent of the loop movement and dynamic correlates with the steric hindrance of the adduct [39,66] (Figure 4). The destabilization of this protein region triggers then the recognition of the alkylated protein by degradation pathway.

### 3.4. Biotechnological Applications of an Engineered SsOGT—the H^5^ Mutant

As described in Section 1.2, the introduction of the SNAP*-tag* technology enabled a wide in vivo and in vitro labelling variety for biological studies by fusing any protein of interest (POI) to this *protein tag* [67]. However, being originated from hMGMT, the extension to extremophilic organisms and/or harsh reaction conditions is seriously limited.

By following the same approach used for the hMGMT as Kai Johnsson [26,27,28,29,30], an engineered version of *Ss*OGT was produced [52,59]. This protein, called *Ss*OGT-H^5^, contains five mutations in the helix-turn-helix domain, abolishing any DNA-binding activity [52]. In addition, a sixth mutation was made—in the *active site loop*, where serine residue was replaced by a glutamic acid at position 132 (S132E). This modification increased the catalytic activity of *Ss*OGT [52,59], as it was observed in the engineered version of the hMGMT during the SNAP*-tag* development [26]. *Ss*OGT-H^5^ shows slightly lower heat stability in respect to the wild-type protein (Table 3), whereas the resistance to other denaturing agents is maintained. Moreover, *Ss*OGT-H^5^ is characterised by a surprisingly high catalytic activity at lower temperatures, keeping the rate of reaction to the physiological ones (Table 3) [52,59]. These characteristics make this mutant a potential alternative to SNAP*-tag* for in vivo and in vitro biotechnological applications. The stability against thermal denaturation allowed Miggiano and co-workers to obtain the structure of the protein after the reaction with the fluorescent substrate SNAP-Vista Green, revealing the peculiar destabilization of the *active site loop* after the alkylation of the active cysteine [66].

#### 3.4.1. In vitro Thermostable H^5^-Based Chimeras

The *Saccharolobus* OGT mutant has been firstly tested as *protein tag* fused to two thermostable *S. solfataricus* proteins heterologously expressed in *E. coli*. The chimeric proteins were correctly folded, and the *tag* did not interfere with the enzymatic activity of the tetrameric *S. solfataricus* β-glycosidase (*Ss*βgly) [59], nor with the hyperthermophile-specific DNA topoisomerase reverse gyrase [68,69,70,71,72]. Furthermore, the stability of H^5^ made possible a heat treatment of the cell-free extract to remove most of the *E. coli* proteins and performing the β-glycosidase assay at high temperatures without the need of removing the *tag* [60].

#### 3.4.2. Expression in Thermophilic Organisms Models

As the applicability of the thermostable *tag* under in vivo conditions is very important, the *Ss*OGT-H^5^ was also expressed in thermophilic organisms. The fluorescent AGT assay allows for the detection of the presence of *Ss*OGT-H^5^ both in living cells as well as in vitro in cell-free extracts [59,72]. To assay the activity to *Ss*OGT-H^5^, it was necessary to choose models in which the endogenous AGT activity is suppressed. *Thermus thermophilus* is an *ogt-* species, showing only one *agt* homologue (TTHA1564), whose annotation corresponds to an alkyltransferase-like protein (ATL) [73]. ATLs are a class of proteins present in prokaryotes and lower eukaryotes [74], presenting aminoacidic motifs similar to those of AGTs’ CTD, in which a tryptophan residue replaces the cysteine in the active site [75]. Like AGTs, ATLs use a helix-turn-helix motif to bind the minor groove of the DNA, but they do not repair it as they only recruit and interact with proteins involved in the nucleotide excision repair system [76,77]. 

Although *T. thermophilus* is a natural *ogt* knockout organism, *Sulfolobus islandicus* possesses an *ogt* gene very similar to that of *S. solfataricus*, which was silenced by a Clustered Regularly Interspaced Short Palindromic Repeats (CRISPR)-based technique and then used as a host organism [72].

The fluorescent signal obtained by SDS-PAGE *gel imaging* revealed that *Ss*OGT-H^5^ not only is efficiently expressed in these thermophilic microorganisms, but it also showed that this *tag* was correctly folded and active, demonstrating the fact that *Ss*OGT-H^5^ might be used as an in vivo *protein tag* at high temperatures [59,72]. As is the case with SNAP-*tag* in human cells, the utilization of *Ss*OGT-H^5^ with different fluorescent substrates gives the opportunity to perform a multi-colour fluorescence study (see Table 1), by following a POI inside living “*thermo* cells” at different stages and localization.

#### 3.4.3. The ASL^tag^ System

As most biotechnological processes require harsh operational conditions, the immobilization of very robust enzymes on solid supports is often essential [78]. By definition, an immobilized enzyme is a “physically confined biocatalyst, which retains its catalytic activity and can be used repeatedly” [79]. Protein immobilisation offers several advantages, such as the catalysts’ recovery and reuse, as well as the physical separation of the enzymes from the reaction mixture. Currently, different immobilisation strategies are available, from physical adsorption to covalent coupling [80,81,82,83]. However, all these procedures require purified biocatalysts and suffer from problems related to steric hindrance between the catalyst, the substrate, and the solid support, with increasing of costs and time for the production processes.

**Figure 5:**
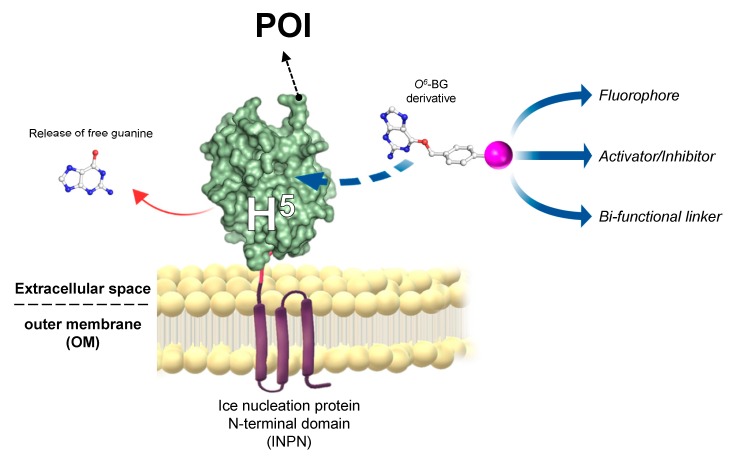
The *novel anchoring and self-labelling protein tag* (ASL*^tag^*) system. A protein of interest (POI) is genetically encoded with the *tag*, which in turn makes it anchored in the outer membrane and accessible for the covalent linkage to a desired chemical group (magenta sphere) by the activity of *Ss*OGT-H^5^ (adapted from [87]).

The introduction of “cell-based” immobilisation systems resulted in a significant improvement and reduces both time and costs of the process. One of the most widely used display strategies is the simultaneous heterologous expression of enzymes and their in vivo immobilisation on the external surface of Gram-negative bacteria cells, by the utilisation of the ice nucleation protein (INP) from *Pseudomonas syringae* [84,85]. Most recently, the N-terminal domain of INP (INPN) was used to produce a *novel anchoring and self-labelling protein tag* (hereinafter ASL*^tag^*). The ASL*^tag^* consists of two moieties, the INPN and the engineered and *Ss*OGT-H^5^ mutant (Figure 5) [86].

The INPN allows an in vivo immobilisation on *E. coli* outer membrane of enzymes of interest and their exposition to the solvent. The significant reduction of the costs related to the purification and immobilization is added to the overcoming of problems related to the recovery of enzymes by simple filtration or centrifugation methods [88]. *Ss*OGT-H^5^, in turn, gives the unique opportunity to label immobilized enzymes with any desired chemical groups (opportunely conjugated to the benzyl-guanine; in magenta in Figure 5) [27,59], dramatically expanding biotechnological applications of this new tool. Depending of the chemical group of choice, modulating the activity of enzymes fused with the ASL*^tag^* can be possible by introducing activator or inhibitor molecules (Figure 5). The ASL*^tag^* system was successfully employed for the expression and immobilization of monomeric biocatalysts, such as the thermostable carbonic anhydrase from *Saccharolobus solfataricus* (*Ss*pCA), as well as the tetrameric *Ss*βgly, without affecting their folding and catalytic activity [86]. Moreover, *Ss*pCA fused to the ASL*^tag^* showed an increase in residual activity of up to 30 % for a period of 10 days at 70 °C [87], representing a huge advantage in pushing beyond reactions in bioreactors and in the reutilization of biocatalysts.

## 4. *Pyrococcus furiosus* and *Thermotoga neapolitana* OGT

To extend the SNAP*-tag* technology to hyperthermophilic microorganisms for in vivo studies, an *O*^6^-alklylguanine-DNA alkyltransferase has been recently characterized from the archaeon *Pyrococcus furiosus* [89]. This extremophilic microorganism was originally isolated from hot marine sediments in Vulcano Island (Italy) [90], with an optimum growth temperature around 100 °C, thus thriving under extremely harsh conditions. Like those of other thermophilic Archaea, its enzymes are extremely thermostable and can be used in various biotechnological applications. For example, DNA polymerase I, also known as *Pfu*DNA polymerase, is one of the most famous and frequently used enzymes from *P. furiosus* because of its high activity, thermostability, and strong 3′-5′ proof-reading activity [91]. The first demonstration of an OGT activity in *P. furiosus* was in 1998, when Margison and co-workers identified a protein of 22 kDa, whose catalytic activity was abolished by the *O*^6^-BG pseudo-substrate. The PF1878 ORF is relative to a protein of 20.1 kDa. From its primary structure, the relative polypeptide seems to be closely related to the MGMT from *Pyrococcus kodakarensis* KOD1 (*Pk-*MGMT) [48,89,92]. The extreme thermostability was confirmed by in vitro biochemical studies on the heterologous expressed and characterized OGT protein from *P. furiosus Pfu*OGT. This enzyme was active on BG-fluorescent substrates, thus allowing the competitive assay with methylated dsDNA. However, the experiments were performed at 65 °C instead of the standard procedure at 50 °C, as described for *Ss*OGT [39,59,60], due to the strong thermophilicity of this enzyme. This behaviour was effectively confirmed by differential scan fluorimetry analysis where the temperature melting (T_m_) of *Pfu*OGT was found to be 80 °C, much higher than that of *Ss*OGT (68 °C) [89]. It is worth noting that, in order to obtain a the sigmoidal melting curve for *Pfu*OGT, a slower heating rate (10 min/°C × cycle) was set up, whereas the T_m_ value measurement is usually performed at 1 min/°C × cycle [93].

*Thermotoga neapolitana* is a hyperthermophilic Gram-negative bacterium of the order of Thermotogales [94,95,96], which are excellent models for genetic engineering and biotechnological applications [97,98,99,100]. The CTN1690 ORF shows a clear homology of the *O*^6^-alkylguanine-DNA-alkyl-transferase. SDS-PAGE *gel imaging* analysis on lyophilized *T. neapolitana* cells incubated with the AGT fluorescent substrate showed a strong fluorescent signal with a molecular weight close to that of *Ss*OGT. The observed molecular weight and, above all, the sensitivity to the *O*^6^-BG derivative, led to the cloning and heterologous expression of the *Thermotoga neapolitana* OGT protein (*Tn*OGT) in *E. coli* [89]. This protein, like most AGTs, has a role in DNA repair, as confirmed by competitive fluorescent assay in the presence of methylated dsDNA. As shown in Figure 3, the IC_50_ value was similar to that obtained for *Ss*OGT. Surprisingly, the enzyme from *T. neapolitana* exhibited a very high activity at low temperatures [89], similar to that possessed by the mutant *Ss*OGT-H^5^ (Table 3) [52,59]. Superimposition analysis between a *Tn*OGT 3D model and the free form of *Ss*OGT (ID PDB: 4ZYE) revealed in both structures the presence of a serine residue in the *active site loop* (S132 in *Ss*OGT, see Figure 2a), which was replaced in *Ss*OGT-H^5^ by a glutamic acid to improve its activity at lower temperatures. Interestingly, some residues are missing in *Tn*OGT that play an important role in stabilizing *Ss*OGT. In particular, the ionic interactions that play a crucial role in the stability of the *Saccharolobus* enzyme at high temperatures, such as the pair R133-D27 [39] and the *K-48 network* [60], are largely replaced by hydrophobic residues in the *Thermotoga* homolog. Evidently, different residues and mechanisms of stabilization may contribute to its exceptional catalytic activity at moderate temperatures and the high thermal stability.

## 5. Future Perspectives

The interest shown from the important insights of this class of small proteins led to novel biotechnological applications [101]. Studies on thermophilic AGTs represent a unique opportunity for structural analysis and, in the case of the *S. solfataricus* protein, for the identification of conformational changes after the trans-alkylation reaction, which are detectable with mesophilic AGTs, as the alkylated form are rapidly destabilized [6]. These results could have a wide impact, especially in medical fields for the design of novel hMGMT inhibitors to be used in cancer therapy [102]. Furthermore, given their small size, thermophilic enzymes are very useful for studying general stabilization mechanisms at high temperatures (as for *Pk*-MGMT and *Ss*OGT), which can then be applied to mesophilic enzymes. Searching for alternative *Ss*OGT homologues was clearly useful, leading to the identification of AGTs that are more resistant to thermal denaturation (*Pfu*OGT) or to enzymes with a higher reaction rate at all tested temperatures (*Tn*OGT).

Concerning biotechnology, the use of a modified hMGMT as *protein tag* opened the possibility to generalise this method—a targeted mutagenesis on a thermostable OGT by following a *rational approach* led to the characterization of *Ss*OGT-H^5^, applicable to in vitro harsh reaction conditions and to in vivo (hyper)thermophilic model organisms. On the other hand, by an *irrational approach* (random mutagenesis) it is also possible to enhance their catalytic activity [103], or modify the substrate specificity of these enzymes, making them active on benzyl-cytosine (*O*^2^-BC) derivatives, such as that which happened for the production of the CLIP-*tag* [104].

This knowledge could be the starting point of developing a new engineered *thermo*-SNAP-*tag* to be employed in particular biotechnological fields, from in vivo studies in (hyper)thermophilic microorganisms (such as the in vivo CRISPR-Cas immune system in *P. furiosus* [105,106]) to industrial processes that require high temperatures or, in general, harsh reaction conditions.

## Figures and Tables

**Table 1:** The use of *protein tags* in some applicative examples.

Applications	FPs	*Affinity Tag*	SNAP-*Tag*	Notes
In vivo imaging	+ ^a^		+	
Substrate utilization	+	−	−	FPs do not need of any substrate for their fluorescence
Emission spectra	±	−	+	FPs are in a limited number with respect to chemical probes
Time-resolved fluorescence	±	−	+	
Multi-colour fluorescence	±	−	+	For FPs, multi-cloning and expression is necessary
In vitro applications	±	±	+	
Variety of chemical group labelling	−	−	+	
Pulse-chase analysis	−	−	+	Fresh synthetized FPs cannot be efficiently quenched
Anaerobic conditions	−	+	+	FPs’ fluorophore formation requires oxygen
Protein purification	+	+	+	Utilization of the GFP-trap matrix
Protein immobilization	+	+	+	Utilization of the GFP-trap matrix
Pull-down experiments	+	+	+	Utilization of the GFP-trap matrix

^a^ +, fully applicable or advantageous; ±, limited applicability; -, not applicable or disadvantageous; FPs = fluorescent proteins.

**Table 2:** Comparison of solvent-accessible surface area and intramolecule contacts.

T_opt_ (°C)	Enzyme (PDB ID)	Total SASA (Å)	Charged Residues Contacts	Intramolecule H-bonds ^a^	References
37	*Escherichia coli* Ada-C (1SFE)	8421.8	74	141	[49]
37	*Mycobacterium tuberculosis* OGT *(*4BHB)	9535.2	56	143	[38]
37	*Homo sapiens* MGMT (1EH6)	8764.3	71	127	[6]
80	*Saccharolobus solfataricus* OGT (4ZYE)	8054.1	94	137	[39]
80	*Sulfurisphaera tokodaii* OGT (1WRJ)	8049.5	124	134	PDB ^b^
80	*Methanocaldococcus jannashii* MGMT (2G7H)	17,770.8 ^c^	N.D.	N.D.	[50]
85	*Pyrococcus kodakaraensis* MGMT (1MGT)	8302.8	111	157	[48]

^a^ Excluding intra-residues H-bonds. ^b^ https://www.rcsb.org/structure/1wrj. ^c^ The structure has been solved by means of NMR explaining the high SASA value.

**Table 3:** Biochemical properties comparison among SNAP-*tag*, *Ss*OGT and the relative H^5^ mutant.

Features		SNAP-*tag*^®^	*Ss*OGT	*Ss*OGT-H^5^
Molecular weight (kDa) ^a^		23.0	17.0	17.0
T_opt_ (°C)		37.0	80.0	75.0
Relative activity	at 25.0°C	80%	25%	**50%** ^b^
	at 37.0°C	100%	45%	**65%**
	at 80°C	-	100%	95%
Catalytic activity at 37 °C (M^−1^ s^−1^)		2.8 × 10^4^	2.8 × 10^3^	**1.6 × 10^4^**
pH_opt_		6.0	7.5	6.0
Thermal stability T_½_ (°C)		6 h (37)	3 h (70)	3 h (70)
Thermal stability T_½_ at 37 °C (h)		6	>24	>24
Additives	NaCl	<0.3 M	>1.0 M	>1.0 M
	EDTA	no	yes	yes
	sarcosyl	no	>0.5%	>0.5%
	DDT	yes	no	no

^a^ Data from [50,57,64]. ^b^ Enhancement with respect to the *Ss*OGT (in bold).

## Abbreviations

AGT	*O*^6^-alkyl-guanine-DNA-alkyl-transferase
CLIP-*tag*	engineered version of SNAP-*tag* active on *O*^2^-BC
FP	auto-fluorescent protein
hMGMT	human *O*^6^-methyl-guanine-DNA-alkyl-transferase
MGMT	*O*^6^-methyl-guanine-DNA-alkyl-transferase
*O*^2^-BC	*O*^2^-benzyl-cytosine
*O*^6^-AG	*O*^6^-alkyl-guanine
*O*^6^-BG	*O*^6^-benzyl-guanine
*O*^6^-MG	*O*^6^-methyl-guanine
OGT	*O*^6^-alkyl-guanine-DNA-alkyl-transferase
SNAP-*tag*	engineered version of hMGMT for biotech purposes
